# Decoupling forest characteristics and background conditions to explain urban-rural variations of multiple microclimate regulation from urban trees

**DOI:** 10.7717/peerj.5450

**Published:** 2018-08-16

**Authors:** Wenjie Wang, Bo Zhang, Lu Xiao, Wei Zhou, Huimei Wang, Xingyuan He

**Affiliations:** 1 Urban Forests and Wetlands Group, Northeast Institute of Geography and Agroecology, Chinese Academy of Science, Changchun, Jilin Province, China; 2 Key Laboratory of Forest Plant Ecology, Northeast Forestry University, Harbin, Heilongjiang Province, China; 3 University of Chinese Academy of Sciences, Beijing, China

**Keywords:** Urban forest types, Cooling humidifying shading effects, Various urban-rural gradients, Redundancy ordination, Variation partitioning

## Abstract

**Background:**

Rapid urbanization in semi-arid regions necessitates greater cooling, humidifying, and shading services from urban trees, but maximizing these services requires an exact understanding of their association with forest characteristics and background street and weather conditions.

**Methods:**

Here, horizontal and vertical air cooling, soil cooling, shading, and humidifying effects were measured for 605 trees from 152 plots in Changchun. Additionally, weather conditions (Tair, relative humidity, and light intensity), forest characteristics (tree height, diameter at breast height (DBH), under-branch height, canopy size, tree density, and taxonomic family of trees) and background conditions (percentage of building, road, green space, water, and building height, building distance to measured trees) were determined for three urban-rural gradients for ring road development, urban settlement history, and forest types. Multiple analysis of variance and regression analysis were used to find the urban-rural changes, while redundancy ordination and variation partitioning were used for decoupling the complex associations among microclimate regulations, forest characteristics, background street and weather conditions.

**Results:**

Our results show that horizontal cooling and humidifying differences between canopy shade and full sunshine were <4.5 °C and <9.4%, respectively; while vertical canopy cooling was 1.4 °C, and soil cooling was observed in most cases (peak at 1.4 °C). Pooled urban-rural data analysis showed non-monological changes in all microclimate-regulating parameters, except for a linear increase in light interception by the canopy (*r*^2^ = 0.45) from urban center to rural regions. Together with the microclimate regulating trends, linear increases were observed in tree density, Salicaceae percentage, *T*_air_, light intensity outside forests, tree distance to surrounding buildings, and greenspace percentage. Redundancy ordination demonstrated that weather differences were mainly responsible for the microclimate regulation variation we observed (unique explanatory power, 65.4%), as well as background conditions (12.1%), and forest characteristics (7.7%).

**Discussion:**

In general, horizontal cooling, shading, and humidifying effects were stronger in dry, hot, and sunny weather. The effects were stronger in areas with more buildings of relatively lower height, a higher abundance of Ulmaceae, and a lower percentage of Leguminosae and Betulaceae. Larger trees were usually associated with a larger cooling area (a smaller difference per one unit distance from the measured tree). Given uncontrollable weather conditions, our findings highlighted street canyon and forest characteristics that are important in urban microclimate regulation. This paper provides a management strategy for maximizing microclimate regulation using trees, and methodologically supports the uncoupling of the complex association of microclimate regulations in fast urbanization regions.

## Introduction

Microclimate changes induced by urbanization in central urban regions (e.g., the heat island effect) have highlighted the importance of multiple microclimate regulation functions performed by trees, including horizontal cooling, air humidification, soil cooling between sunny and shady sites, vertical cooling adjustment, and radiation interception (Sanusi et al., 2016; Yan et al., 2012; Zhang et al., 2017a). Currently, there are three hypotheses to explain the large variations in microclimate regulation of trees present in the same city. One hypothesis focuses on forest characteristics and suggests that the nature of the forest itself largely determines microclimate regulation, and that characteristics such as tree species (Abreu-Harbich, Labaki & Matzarakis, 2015; Sanusi et al., 2017; Shahidan et al., 2010), tree size, and community features (Martini, Biondi & Batista, 2017; Zhang et al., 2017a), or proper configuration of trees (Bajsanski, Stojakovic & Jovanovic, 2016; Calcerano & Martinelli, 2016; Zhao, Sailor & Wentz, 2018a; Zhao, Wentz & Murray, 2017), could maximize urban forest service functions (Kendal, Dobbs & Lohr, 2014; Xiao et al., 2016b; Zhao et al., 2018b). Another hypothesis focuses on weather and suggests that instantaneous weather conditions are important in shaping the microclimate regulatory functions of trees, and that the general tendency is that dry and sunny weather accompanies stronger regulatory functions (Norton et al., 2015; Zhang et al., 2017a). The “background build-up” hypothesis describes background conditions that largely shape microclimate regulation by trees, such as building geography (e.g., street orientation; (Sanusi et al., 2016), street canyon features (e.g., building height, distance to measured trees; (Coutts et al., 2016; Morakinyo et al., 2017; Rahman et al., 2017), land use configurations (building, street, greenspace, and water) and urbanization intensity (Ren et al., 2013; Yahia & Johansson, 2014). To date, the contributions of each of the various factors affecting tree microclimate regulation are not well-defined, and utilization of statistical analysis methods such as redundancy ordination and variation partitioning analysis may benefit our understanding of the underlying mechanisms of variation in microclimate regulation (Legendre & Legendre, 1998; Wang, Wang & Chang, 2017a).

Rapid urbanization has occurred in China, with an urbanization percentage increase from 17.92% in 1978 to 58.52% in 2017, and 813.47 million people live in urbanized regions today (China-National-Statistics-Bureau, 2017). Urbanization changes the local climate, including urban thermal conditions, net radiation, and energy balance, and wind field and water balance (Ren et al., 2013; Wilmers, 1997; Yahia & Johansson, 2014). Urbanization also results in changes to background land use configuration, street canyon and building arrangement (Zhang et al., 2017b; Zhang et al., 2015). The effects of urbanization on nearby forests include changes to species diversity and carbon sequestration in urban vegetation (Lv et al., 2016; Xiao et al., 2016a), soil carbon sequestration and turnover (Zhai et al., 2017), and alterations of soil nutrient and physiochemical properties (Wang et al., 2018a; Zhou et al., 2017). In these studies, urban-rural gradients were classified by urban settlement history, ring road development, and land use. In the process of urban development, some forest types can be defined as roadside forests (RF), which are found mainly in street surroundings, and affiliated forests (AF), which are found mainly in schools, institutes, universities, and residential areas. These urban forests increase in proportion with urbanization, while landscape forests (LF) in parks and botanical gardens and ecological and public welfare forests (EF) that are distributed among the urban-rural integration areas (farmland protection forests and other ecological protection forest for water supply areas), decrease (He et al., 2004; Lv et al., 2016). Urbanization is a process of changes in forest characteristics, microclimate conditions, background arrangement of land use, buildings and streets; however, patterns of microclimate regulatory functions from urban forests have not be studied in different urbanization regions such as those on different urban-rural gradients (Wang et al., 2018b).

In this study, a large field survey was carried out in the major city of Changchun in northeastern China, which included five ring-road developments, an urban settlement history of over 100 years, and vigorous development of urban forests and green spaces (Ren et al., 2013; Zhou et al., 2017), to test the hypotheses that the microclimate regulatory functions of urban trees are location-dependent across different urban-rural gradients, and that forest characteristics, surrounding street canyon and land use backgrounds, and weather conditions are responsible for these microclimate regulation variations. To address these hypotheses, we want to answer the following four questions:

(1) What are the main differences in the shading, cooling, and humidifying effects of urban trees in different regions of the urban-rural gradient? What are the concurrent changes in forest characteristics, weather conditions, and street canyon and land use backgrounds?
(2) Which factors (weather, forest, or background conditions) are responsible for microclimate regulatory variations? What recommendations can be made for the management of urban forests?

## Materials & Methods

### Study site

**Figure 1:**
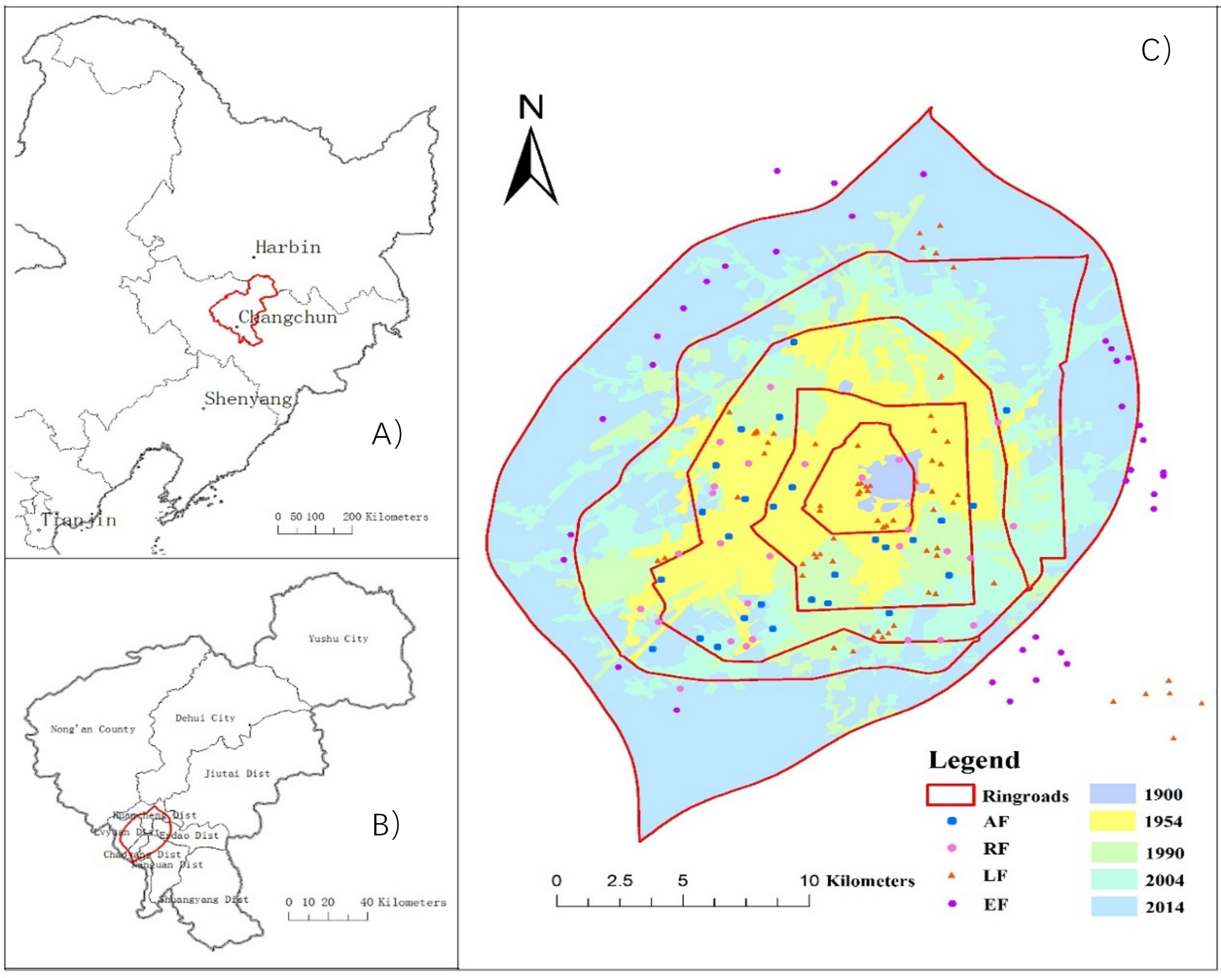
Location of and sample sites in the study area. (A) The map of Northeast China, (B) the map of Changchun administrative region and (C) the map of study area, in which the shading represented the regions of different human settlement time and the red lines indicated the different ring road boundaries (from inner to outer matched the ringroad 1st to 5th regions). The map was created using ArcGIS 10.3 (Esri, Redlands, CA, USA; http://www.esri.com/software/arcgis).

The study sites were in Changchun (43°05′–45°15′N and 124°18′–127°02′E), which is the capital city of Jilin Province in northeast China. The climate is a continental monsoon climate in the North Temperate Zone. The annual average temperature was 4.8 °C, and annual precipitation was 567 mm (Wang et al., 2018a; Zhai et al., 2017; Zhang et al., 2017b). Our study area was primarily located within the first to fifth ring roads of Changchun (Fig. 1), which included an area of around 500 km^2^. The main trees in the urban forests included *Populus* and *Salix* species*, Picea* spp. (Pinaceae), *Syringa* (Oleaceae) and *Pinus* spp. (Pinaceae) (Ren et al., 2015). A stratified sampling method was adopted to allocate sampling plots that provided a balanced survey across all the urban forests in the city. A total of 605 trees from 152 plots were surveyed for this study. The distribution of plots is shown in Fig. 1 and was classified into gradients related to ring road, urban history, and forest type (Table 1). Some trees measured outside the fifth ring road belong generally to ecological welfare forests (mainly farmland shelterbelt forests) and landscape forests (located in Jingyue Park). Healthy trees that were pest-free, had no stent support, and had a regular crown shape were selected for measurement.

**Table 1:** Trees measured in urban-rural gradient regions surveyed in this study.

Classification	Region	Tree measured	Plot number
Ring road-related urban rural gradient	1st ring road region	64 trees	16 plots
	2nd ring road region	137 trees	35 plots
	3rd ring road region	177 trees	45 plots
	4th ring road region	32 trees	8 plots
	Outside 4th ring road region	195 trees	48 plots
Urban-history urban rural gradient	114-yr-old region	20 trees	5 plots
	60-yr-old region	192 trees	48 plots
	24-yr-old region	114 trees	30 plots
	10-yr-old region	68 trees	17 plots
	New-urbanized region (0-yr)	211 trees	52 plots
Forest-type urban-rural gradient	RF roadside forests	104 trees	26 plots
	AF affiliated forests	96 trees	25 plots
	LF landscape forests	265 trees	67 plots
	EF ecological welfare forests	140 trees	34 plots
Total measured		605 trees	152 plots

### Measurement of microclimate and forest compositional and tree size parameters

**Figure 2:**
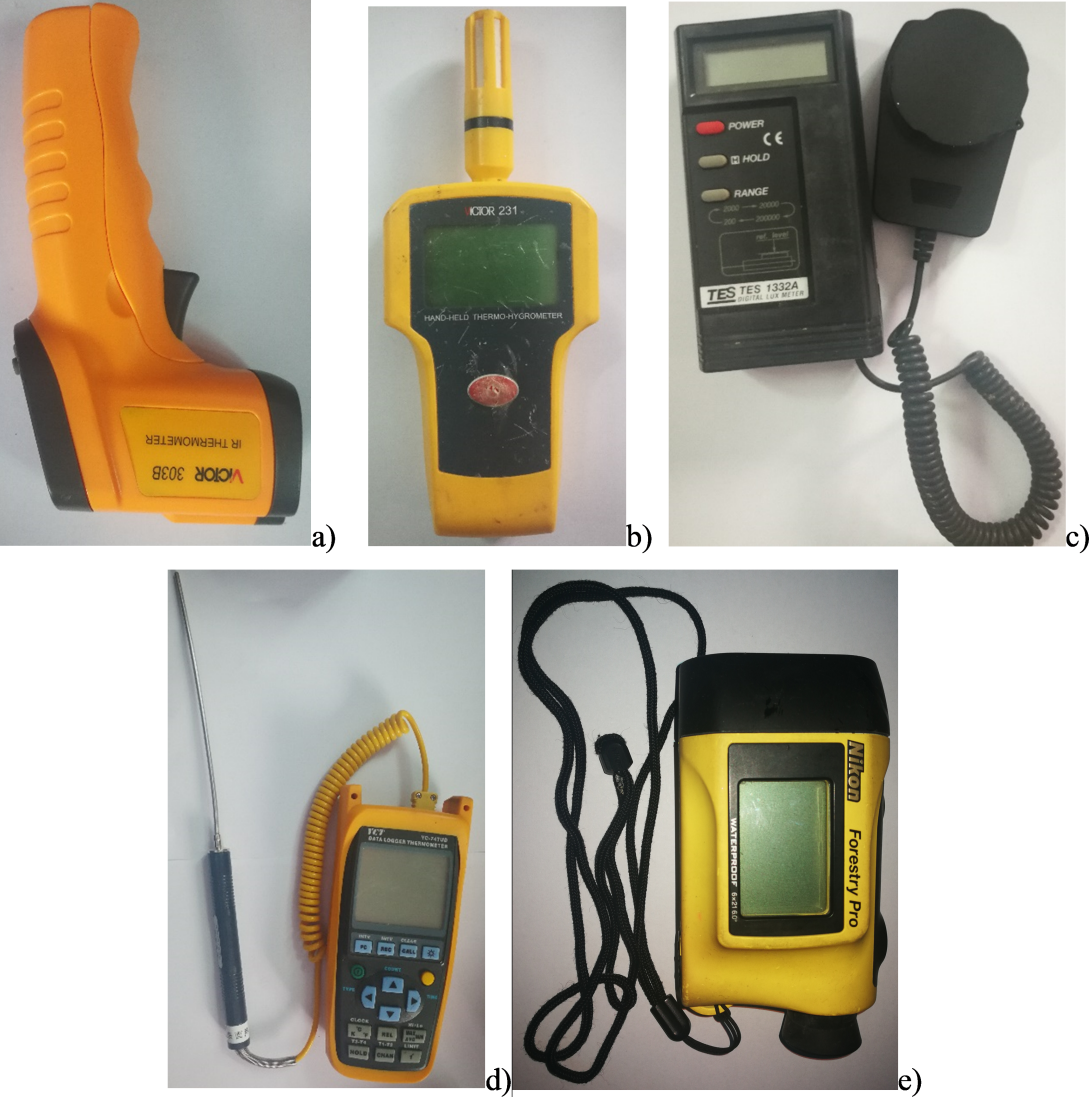
The equipment used in this study. (A) Canopy temperature; (B) air temperature and relative humidity; (C) light intensity; (D) soil temperature; (E) tree height. Pictures by Wenjie Wang.

We measured tree height and under-branch height (m) of the studied trees using a NIKON forestry PRO laser tree height meter (Nikon Corporation, Tokyo, Japan) (Fig. 2). The diameter at breast height (DBH) was measured in centimeters using a regular soft tape measure placed around the tree 1.3 m above the ground (as a rule for the tree census, diameter was usually measured at this height). Canopy size was measured as the projection area of the canopy using an elliptic area formula (m^2^) using the east–west and north-south radii. For all these measurements, at least four replicates were included for the main tree species. Species, genus, and family names were recorded for all trees studied. These data were used to find differences between the ring road and urban historical regions, and between different forest types. The compositional differences were described as the family percentage at each gradient or forest type with reference to the total trees surveyed.

**Figure 3:**
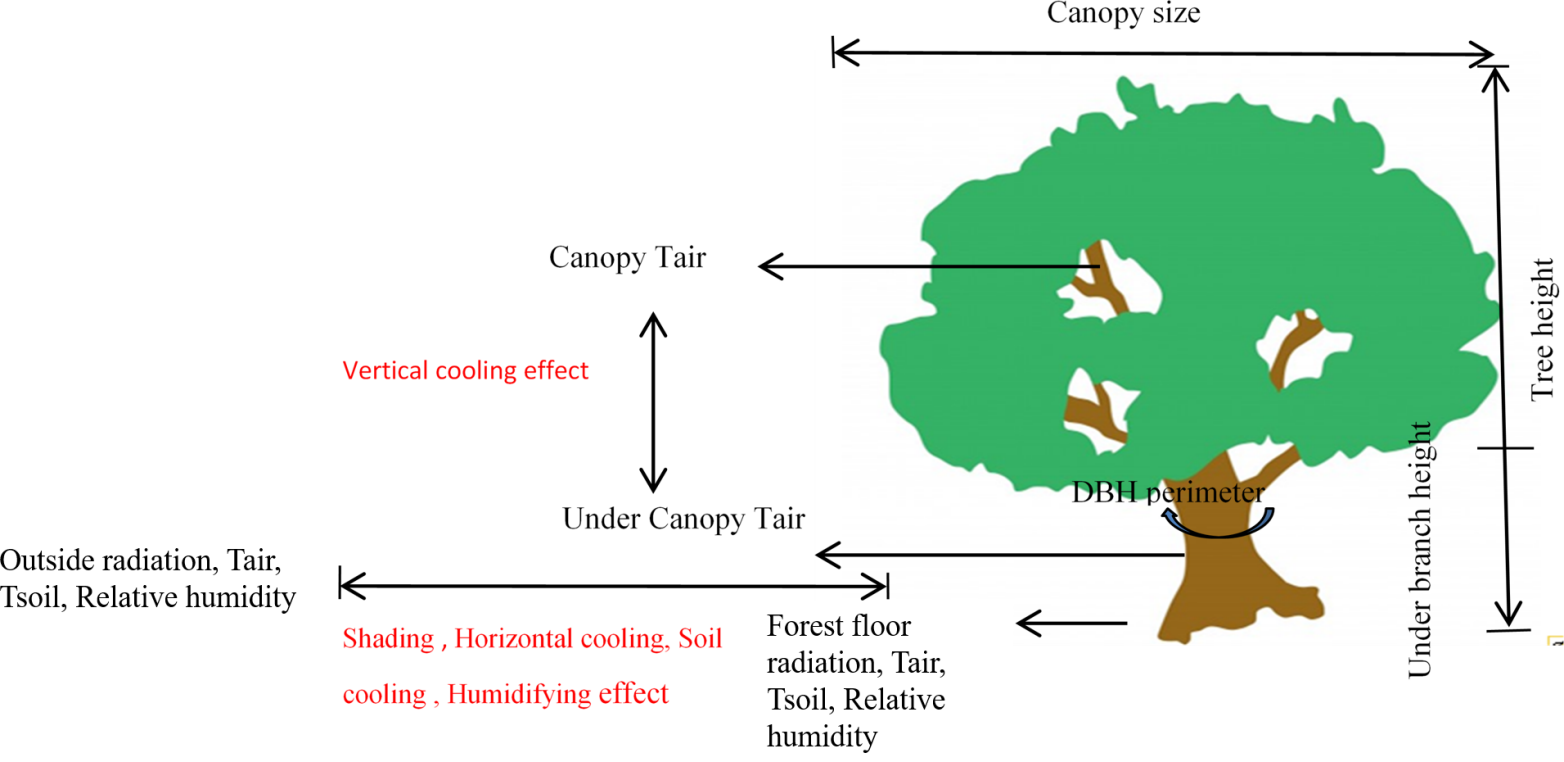
Schematic of the various microclimate parameter measurements and the relative position of measured trees. The shading, horizontal cooling, vertical cooling, soil cooling and humidifying effects from urban trees were calculated from these measurements. *T*_air_ and *T*_soil_ are air and soil temperature, respectively. The distance from the canopy shade to forest outside used for microclimate regulation calculation was approximately 10 m.

Light intensity, air temperature, relative humidity, and soil temperature were measured in open areas outside of forests during the plot inventory survey (the equipment for these measurements was in Fig. 2). Canopy surface temperature and under-canopy temperature, which was assessed at the lowest part of the canopy, were measured with an infrared hand-held temperature measurement gun (303b Victor; Shenzhen Victory Hi-tech Co., Ltd., Shenzen, Guandong, China) (Zhang et al., 2017a). Both the middle and lowest canopy temperatures were measured with the same equipment to guarantee data precision. The measurement sites are shown in Fig. 3 and these data were used for computing the cooling, shading, and humidifying effects of urban trees. Air temperature (*T*_air_) and relative humidity (RH) were measured with a handheld temperature and humidity meter (Victor231; Shenzhen Victory Hi-tech Co., Ltd., Shenzen, Guandong, China) in the shade, approximately 10 m away from the canopy shade (outside of the forest). Similarly, solar light intensity was measured with a digital illuminance meter (Tes-1330a; Tai Electronic Industry Co., Ltd., Shenzen, China), and soil temperature was measured with a thermorecorder (YC-747UD; YCT, Guangzhou, China) equipped with a needle thermometer (NR81530; Shangjiayiqi, Shanghai, China) that was inserted to a soil depth of 5 cm. These data were used to calculate microclimate regulatory functions in the next section, and the light outside of the forest (Light), *T*_air_, and RH were used as weather conditions in this study. In the field measurements, at least three replicate measurements were taken in each plot to produce reliable microclimate regulation data.

### Measurement of street canyons, building features, and land use configurations

During the field survey, the trees used for measurement collection were precisely marked in the Baidu map (https://map.baidu.com/). The background street canyons, surrounding building features, and land use configuration were measured from street-view pictures and the earth-view Baidu map. For each measurement site, we used the distance measuring tool in the Baidu map to draw a 200 m × 200 m square with the measuring site at the center, and then the relative percentage of building area, road area (all roads, streets, and other impervious surfaces like parking lots, square plazas, sport fields, etc.), green space area (forests, grasslands, farmlands, etc.), and water area (rivers, wetlands or lakes) were estimated for each site. In the earth-view mode of the Baidu map, we measured the distance to the nearest building from each measured tree, and the distance to buildings in four cardinal directions from each measured tree, using the distance-measuring tool that is freely available in the Baidu map program. The distances in four directions were averaged as the mean distance of buildings to the measured trees, and the shortest distance was recorded as the closest distance between the buildings and the measured trees. In the street-view mode of the Baidu map, the building height nearest to the measured trees was measured by assuming a 2.8-m average height per floor of the building, according to the national standards for designing codes of residence buildings (Ministry-of-Housing & Urban-rural-Development-of-China, 2012). Figure A1 shows this measuring process.

### Calculation of microclimate shading, cooling, and humidifying regulation

As shown in Fig. 3, the shading effect was represented by the total light intercepted by the canopy: [ΔE(*k*_Lux_)], ΔE = outside light − undercanopy light; the shading degree (%) was calculated as a percentage: shading degree (%) $= \frac{\Delta \mathrm{E}}{\text{outside light}} \times 100\text{%}$. Horizontal cooling was represented by Δ*T*_1_(°C), Δ*T*_1_ = outsideforest  *T*_air_ − forestfloor   *T*_air_; the vertical cooling difference was represented by Δ*T*_2_(°C), Δ*T*_2_ = Canopy *T*_air_ − undercanopy *T*_air_; the soil cooling difference was represented by Δ*T*_3_(°C), Δ*T*_3_ = outside forest *T*_soil_ − forest floor *T*_soil_; the humidifying effect was represented by ΔRH(%), ΔRH = forest shade RH-outside forest RH (Zhang et al., 2017a; Zhang, Lv & Pan, 2013). For horizontal cooling, soil cooling, shading effects, and humidifying effects, Δ*T*_1_, Δ*T*_3_, ΔE, shading degree (%), and ΔRH indicated the changing rate in temperature, air humidity, and light intensity at a given distance (10 m) from the canopy shade. To compare the differences throughout the urban-rural gradient, the larger differences in microclimate regulation indicated a larger attenuation rate and a shorter effect distance from the canopy shade (Fig. 3).

### Data analysis

To assess the changes of all tested parameters throughout urban-rural gradients, linear regressions between urban-rural gradients (digitized as 1 to 5) and microclimate regulation, forest characteristics, and weather and background conditions, were performed with JMP10 (SAS, Cary, NC, USA), and both separated data (ring road, urban history, and forest type urban-rural gradients) and pooled data were analyzed. For ease in the regression analysis, we assigned the numbers of 1, 2, 3, 4 and 5, respectively, to the first, second, third, fourth, and outside ring road regions; to regions that were 114, 60, 24, and 10 years old or newly urbanized (0 years); and to each type of forest including roadside forests, affiliated forests, landscape forests, and ecological welfare forests. Included as an appendix to this paper, multiple analysis of variance (MANOVA) and post-hoc multiple comparisons were also performed at different ring road, urban history, and forest type urban-rural gradients

The redundancy ordination analysis (RDA) was used to graphically represent the associations between microclimate regulating function, weather conditions outside the forest, forest characteristics, and background conditions. The basic idea for RDA has been described previously (Wang et al., 2017b). Canoco 5.0 (Biometris, Wageningen, Netherlands) was used for the RDA analysis, and the variation partitioning procedure ‘Var-part-3groups-Simple-effects-tested’ was used for partitioning the microclimate regulation variation into three groups of factors: weather conditions, forest characteristics, and background conditions. Conditional term effects and simple term effects were also used to compare the relative importance of each of the tested variables in explaining the variations in microclimate regulation. To statistically check the significance of the parameter selections, both regular *p*-value and Bonferroni-corrected *p*-values were calculated in the forward selection of parameters, for both conditional and simple term effect analyses.

## Results

### Microclimate regulation differences

**Figure 4:**
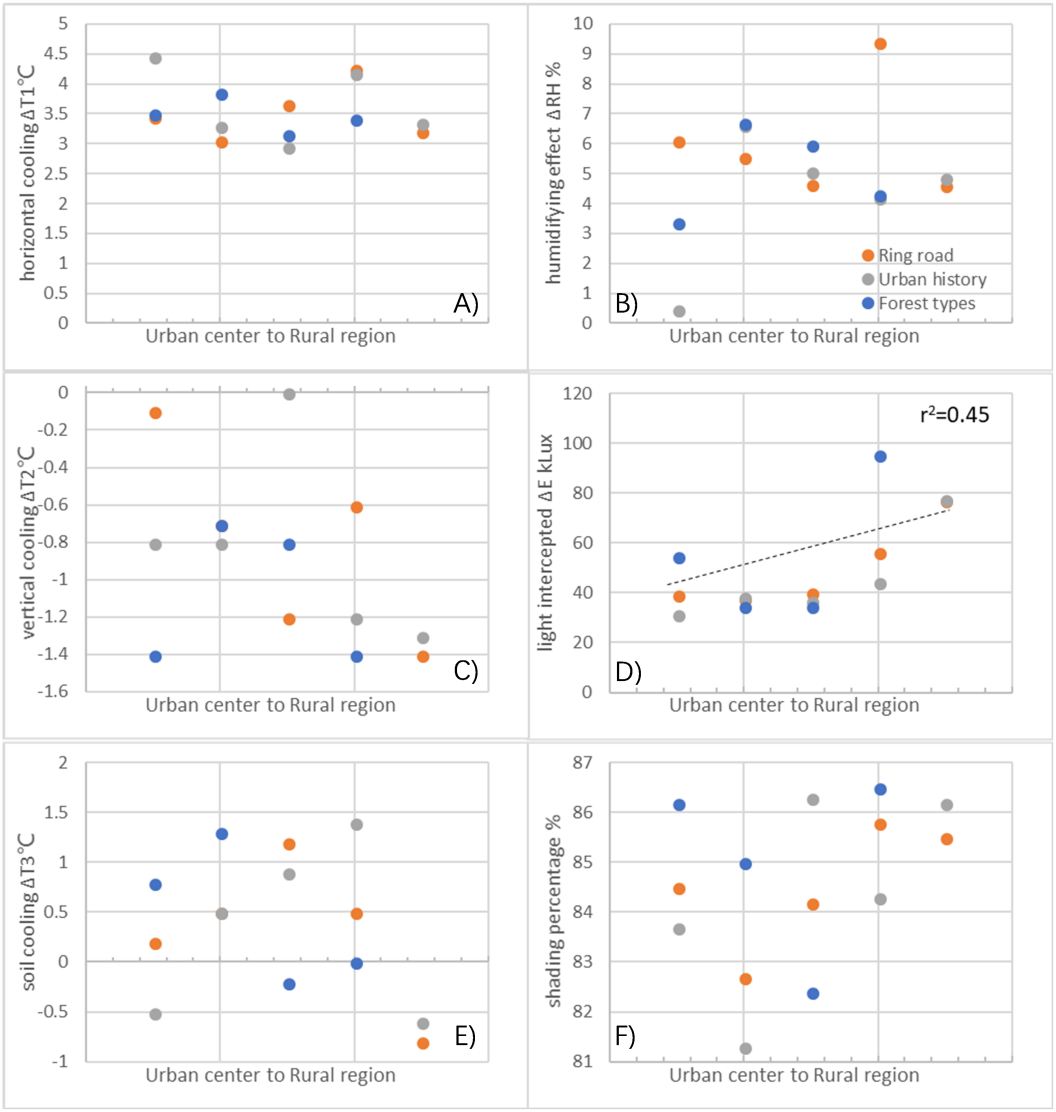
Differences of microclimate regulation variations in trees from three urban-rural gradient regions. (A) Horizontal cooling; (B) humidifying effect; (C) vertical cooling; (D) light interception amount; (E) soil cooling; (F) shading percentage. Dashed line in the figures indicates significant change in the regression analysis (*p* < 0.05).

In a gradient from the urban center to rural regions, pooled data did not reveal linear changes in Δ*T*_1_, Δ*T*_2_, or Δ*T*_3_ (Fig. 4), although unpooled data revealed significant decreases in Δ*T*_2_ in the ring road-related gradient, and significant Δ*T*_3_ in the ring road and forest-type gradients (Fig. A2). All the data showed that under forest shade, *T*_air_ was lower than those in the open sites, with a range of 3.05 to 4.46 °C in different urban regions (Table A1). The vertical cooling difference (Δ*T*_2_) showed that canopy air temperature was usually lower than undercanopy air temperature (highest at 1.4 °C). For forest types, the RF and the EF had the highest Δ*T*_2_, about 2-fold higher than those in the AF (−0.7 °C; Table A1). The soil cooling difference (Δ*T*_3_) showed that soils outside of forest coverage had a higher temperature than those under the canopy shade; however, contradictory results were also observed in some regions (Table A1).

The humidifying effect (ΔRH) regression analysis of pooled (Fig. 4) and unpooled data (Fig. A2) showed no linear changes from the urban center to rural regions; however, ΔRH significantly differed between ring road regions, forest types, and urban historical regions (*p* < 0.05). Humidifying effects were observed in all tested areas and ΔRH ranged from 4.62% to 9.41% in different ring road regions, 0.46% to 6.65% in different urban historical regions, and displayed a 1.9-fold variation in different forest types (Table A1).

Unlike the other parameters mentioned above, shading effect-related regression analysis of pooled data (*r*^2^ = 0.45, Fig. 4) and unpooled data (*p* < 0.001, Fig. A2) showed a linear increase in ΔE from the urban center and rural regions. Peak ΔE was 77.1 kLux in outside ring road regions, while the highest shading function was also found in rural regions related to forest types and urban history, e.g., EF’shading effects were 95.3 kL_ux_ and 86.5%, and new settlement regions (0 years) had the shading effects of 76.5 kL_ux_ and 86.2% (Table A1).

### Forest characteristics: tree size, density, and composition

**Figure 5:**
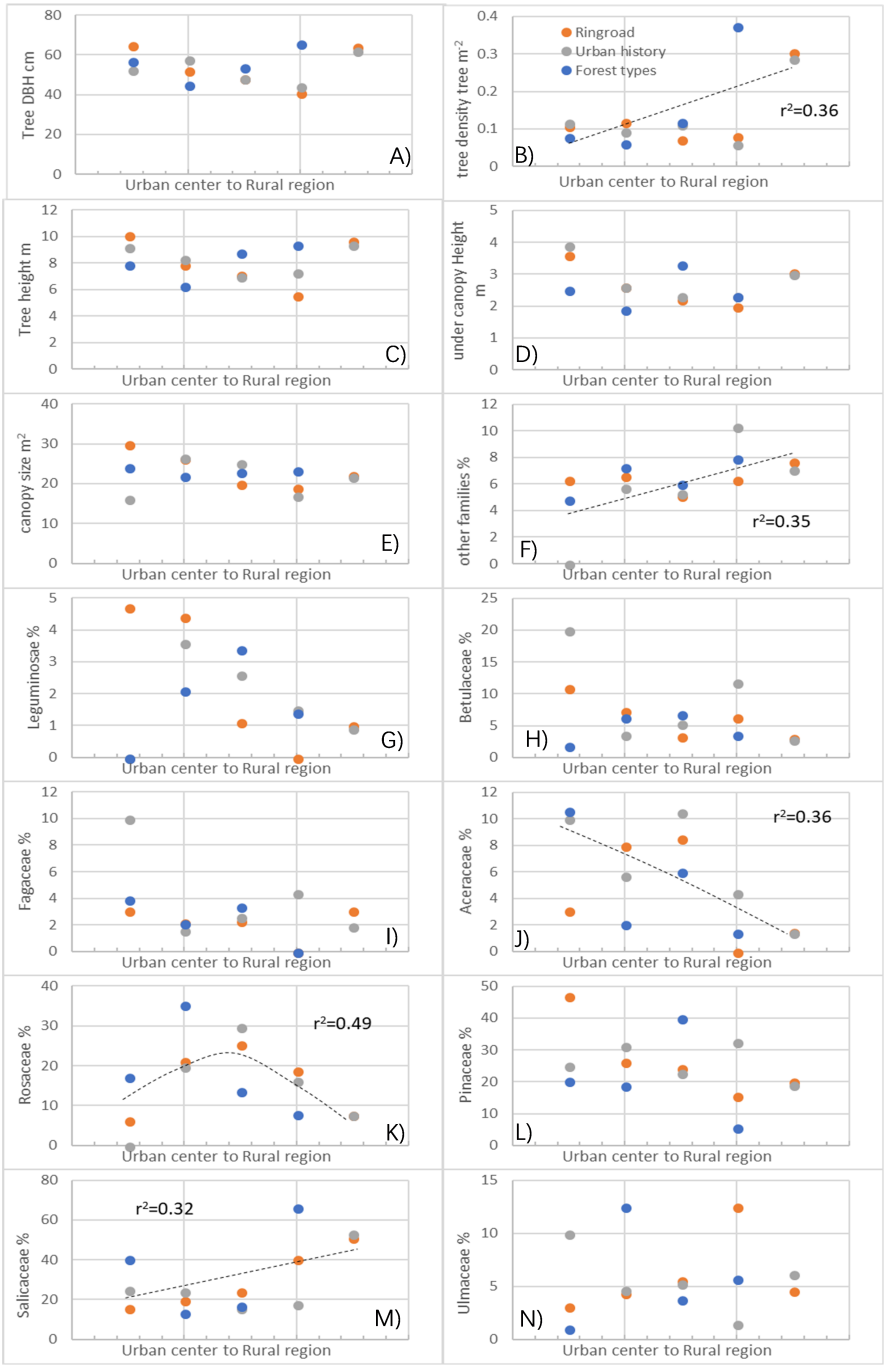
Differences in forest characteristics (i.e., tree sizes, density and compositional traits) where the microclimate regulation by trees was measured in different urban-rural regions. (A) Tree diameter; (B) tree density; (C) tree height; (D) under canopy height; (E) canopy size; (F) other family percentage in the forests; (G) Leguminosace percentage in the forests; (H) Betulaceae percentage in the forests; (I) Fagaceae percentage in the forests; (J) Aceraceae percentage in the forests; (K) Rosaceae percentage in the forests; (L) Pinaceae percentage in the forests; (M) Saliaceae percentage in the forests; (N) Ulmaceae perccentage in the forests. Dashed lines in the figures indicate significant changes in the regression analysis (*p* < 0.05).

Neither pooled (Fig. 5) nor unpooled data (Fig. A3) from the three urban-rural gradients showed significant linear changes in tree size parameters. However, tree size parameters differed significantly in the tested ring road, urban history, and forest type regions (Table A2). Moreover, these differences were even more evident in the assessment of tree height, DBH, and under-branch height than in canopy size. Tree height and DBH ranged from 5.6 m to 10.1 m and 41.1 cm to 65.6 cm in different regions, respectively (Table A2).

Tree density significantly increased from urban to rural regions (*p* < 0.05), as shown in the pooled (Fig. 5) and unpooled data (Fig. A3) from three urban-rural gradient regions. In different ring road regions, tree density increased from about 0.1 trees/m^2^ in urban centers to 0.3 trees/m^2^ in rural regions, and a similar increase was found in urban history and forest type urban-rural gradients (Table A2).

In family compositional traits, there was a general increase in the Salicaceae percentage (*r*^2^ = 0.32) and the percentage of other tree families (*r*^2^ = 0.35), but a decrease in the Aceraceae percentage (*r*^2^ = 0.36) was observed (Fig. 5). However, the Rosaceae percentage in forests was peaked in regions with medium urbanization (*r*^2^ = 0.49; Fig. 5).

### Weather and background conditions

**Figure 6:**
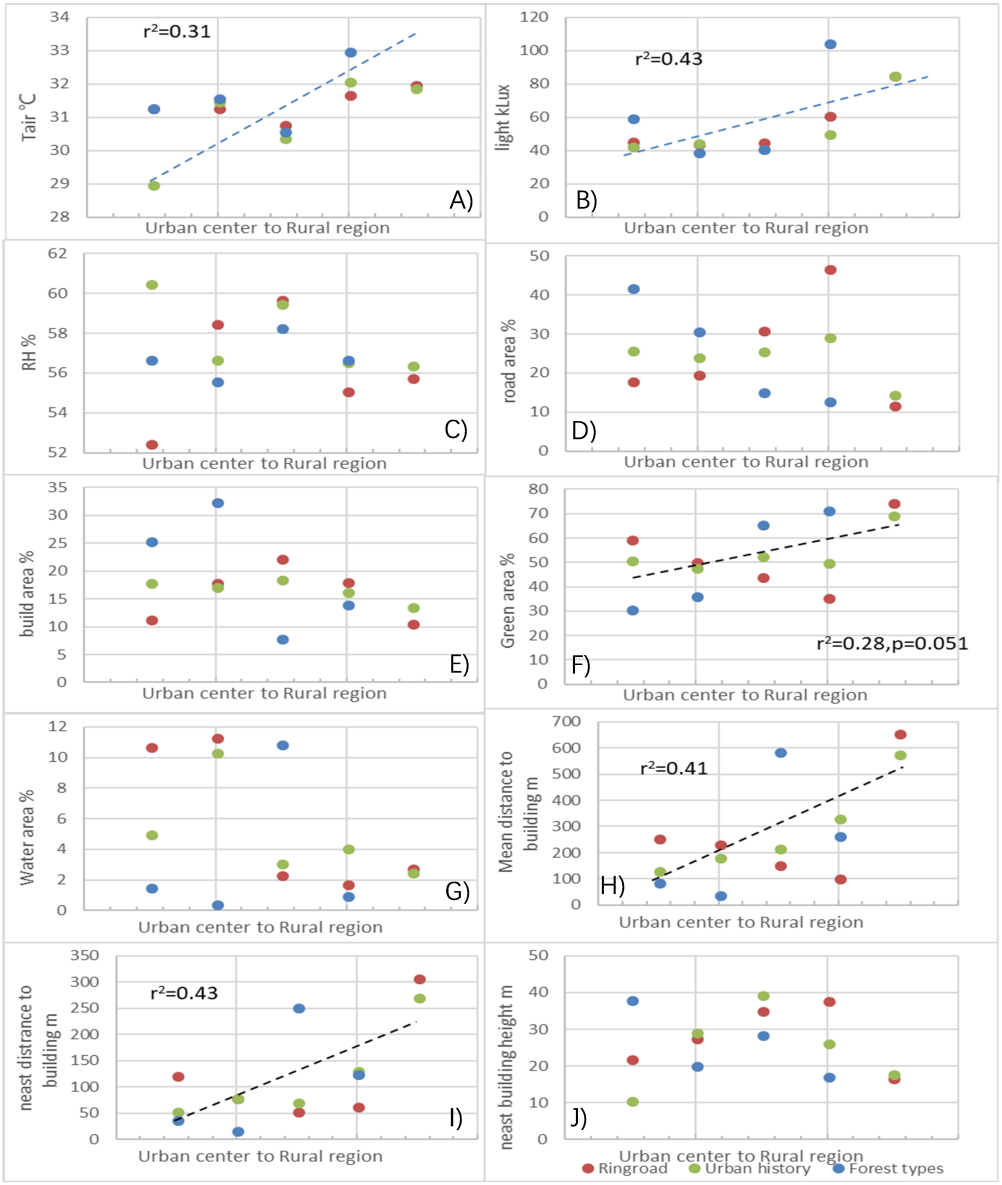
Differences in background conditions where the microclimate regulation by trees was measured in different urban-rural regions. (A) Air temperature; (B) light intensity; (C) air humidity; (D) road area in percentage; (E) building area in percentage; (F) green area in percentage; (G) water area in percentage; (H) mean distance to building from the measured trees; (I) nearest distance to building from the measured trees; (J) nearest building height from the measured trees. Dashed lines in the figures indicate significant changes in the regression analysis (*p* < 0.05).

Instantaneously-measured weather conditions were significantly different between different ring road regions, urban historical regions, and forest types (Table A3), and the linear regression of pooled data showed a linear increase in *T*_air_ (*r*^2^ = 0.31) and light intensity (*r*^2^ = 0.43) from the urban center and rural regions (Fig. 6). Different urban-rural gradients also showed different trends from the urban center to rural regions (Fig. A4). The highest RH was found in the third ring road region (59.7%), while the lowest was observed in the first ring road region (52.5%) (*p* < 0.05). The RH for urban historical regions ranged from 56.4% to 60.5%, and the RH for different land uses ranged from 56.7% to 58.3% (Table A3).

The land use configuration percentage, building height, and distance to measured trees showed monological changes from the urban center and rural regions (Fig. 6). Pooled data analysis indicated a significant linear increase in green space percentage (*r*^2^ = 0.41), nearest distance to surrounding buildings from the measured trees (*r*^2^ = 0.43), and mean distance to buildings in the cardinal directions (*r*^2^ = 0.41; Fig. 6). Specifically, the area percentage of road and greenspace were 11.9% to 46.9% and 30.9% to 74.7% at different regions, respectively (Table A3). Water comprised the smallest percentage of the occupied area, and on average the configuration percentage was 0.4% to 10.8% in different regions (Table A3).

### Redundancy ordination and variation partitioning

**Figure 7:**
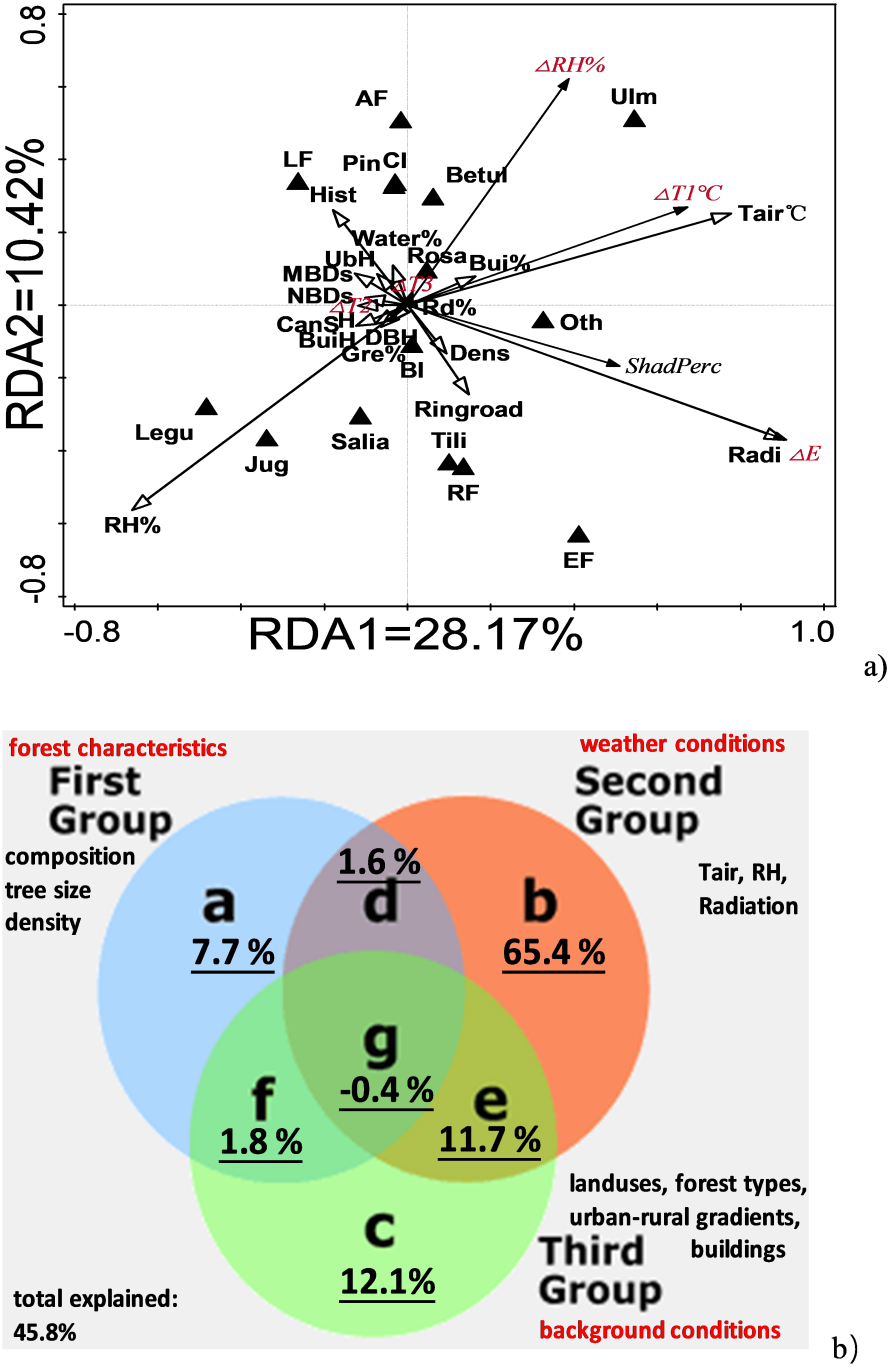
Redundancy ordination between microclimate regulation functions, background conditions, forest characteristics and weather conditions. (A) Ordination results and (B) RDA-related variation partitioning between background conditions, weather conditions and forest characteristics. Abbreviations used in figure: microclimate regulation functions: Δ*T*_1_, Δ*T*_2_, Δ*T*_3_, Δ*E*, shading (%) and ΔRH. Background conditions: distance to nearest building (NBDs); height of nearest building (BuiH), mean distance to buildings (MBDs); road percentage, (Rd%); green space percentage (Gre%); build percentage (Bui%); water percentage (water%); forest types (AF, LF, EF and RF); urban history (Hist); and ring road region (Ring road). Weather conditions outside forests: *T*_air_, RH, Radiation. Forest characteristics: DBH, Tree height (H), canopy size (Can S), under branch height (UBH), tree density (Dens), broadleaf (BL), conifer leaf (CL), and forest composition percentage of the families Ulmaceae (Ulm), Pinaceae (Pin), Betulaceae (Betul), Leguminosae (Legu), Salicaceae (Salia), Juglaceae (Jug), Rosaceae (Rosa) and others (Oth).

Redundancy analysis visually shows the complex associations among microclimate regulation, forest characteristics, weather conditions outside the forests, and background conditions (i.e., land use, urbanization, and street canyon features; Fig. 7A).

A higher percentage of Ulmaceae, and a lower percentage of Leguminosae, Juglaceae, and Salicaceae, were associated with a larger Δ*T*_1_ and higher *T*_air_ but lower air humidity. Moreover, an increased percentage of total area devoted to buildings, but with lower building height, was associated with stronger horizontal cooling. Compared to Δ*T*_1_, the arrow length for vertical canopy cooling (Δ*T*_2_) and soil cooling (ΔT_3_) were much shorter, indicating their contributions to microclimate regulation were much smaller than that of horizontal cooling (Δ*T*_1_; Fig. 7A). Shading amount (ΔE) and shading percentage (%) were closely related, and both were positively correlated with light intensity outside the forest (Fig. 7A). The shading effects were positively related to tree density, but negatively related to the distance to neighboring buildings. In general, shading effects increased from the urban center (first ring road region or regions with a long history) to the rural regions (newly urbanized regions; Fig. 7A), which confirmed our linear regression analysis in Fig. 4, i.e., linear decreases from the urban center to rural regions in shading effects. The higher percentage of Ulmaceae, but lower percentage of Leguminosae, Juglaceae, and Salicaceae, were consistent with the higher humidification and shading effects. ΔRH was higher in forests that had a higher percentage of Ulmaceae and a lower percentage of Leguminosae, Juglaceae, and Salicaceae (Fig. 7A). In general, an increased percentage of area covered by buildings was in line with the higher horizontal cooling and humidification effects, and a shorter distance between trees and buildings in the rural regions were accompanied by the higher shading effects. Vertical cooling (Δ*T*_2_) positively correlated with the distance to the nearest building, tree height (total and underbranch), and canopy size (Fig. 7A).

Both simple and conditional term effect models indicated that radiation (24.6%), *T*_air_ (9.5–18.2%), and RH (2.2–15.7%) could explain most of the variation in microclimate regulation (Table 2) when compared to those that used forest characteristics (<1.5% for each parameter) and background conditions (<3.0% for each parameter). The forest characteristics of tree size (height, canopy size) and tree type composition (Ulmaceae, Salicaceae, etc.) had significant explanatory power for variations in microclimate regulation (*p* < 0.05). The background conditions, including street canyon features (distance to building, building height), land use configuration (build area percentage), and urban-rural gradients (forest types, ring road development, and urban historical regions) had significant explanatory power for the microclimate variations, too (*p* < 0.05; Table 2).

**Table 2:** RDA-related simple term and conditional effects of the complex associations between microclimate regulation functions, background conditions and forest characteristics.

Group	Parameter name	Explains %	pseudo-F	P	P(adj)
Simple term effects (only significant ones listed here)					
Weather conditions	Radiation (*k*_Lux_)	24.6	190	0.002	0.064
	*T*_air_ (°C)	18.2	130	0.002	0.064
	RH (%)	15.7	109	0.002	0.064
Background conditions	EF forest type	3.0	17.9	0.002	0.064
	LF forest type	2.8	16.9	0.002	0.064
	Ring road	2.1	12.5	0.002	0.064
	History gradient	1.9	11.1	0.002	0.064
	Build-area (%)	1.6	9.3	0.002	0.064
	Mean build distance	0.8	4.9	0.004	0.128
	RF forest type	0.8	4.8	0.004	0.128
	Water area (%)	0.6	3.8	0.008	0.256
	Build distance to nearest tree (m)	0.6	3.4	0.01	0.32
	Green space area (%)	0.5	2.9	0.026	0.832
Forest characteristics	Canopy size (m^2^)	1.5	8.7	0.002	0.064
	Height (m)	1.5	8.7	0.002	0.064
	Underbranch height (m)	1.1	6.6	0.002	0.064
	DBH perimeter (cm)	1	6.1	0.002	0.064
	Salia family	1	6	0.004	0.128
	AF forest type.	0.9	5.2	0.002	0.064
	Ulm family	0.7	3.9	0.008	0.256
	Cl leaf type	0.6	3.8	0.006	0.192
	Bl leaf type	0.6	3.8	0.01	0.32
	Pin family	0.6	3.6	0.008	0.256
	Tree density	0.5	3	0.03	0.96
Data statistics	Microclimate conditions	58.5/100			
	Forest characteristics	9.1/15.6			
	Background conditions	15.6/26.7			
Conditional term effects (only significant ones listed here)					
Weather conditions	Radiation (_kLux_)	24.6	190	0.002	0.064
	RH (%)	9.5	83.8	0.002	0.064
	*T*_air_ °C	2.2	19.9	0.002	0.064
Forest characteristics	Height (m)	1.4	13.1	0.002	0.064
	Salia family	1.6	15.7	0.002	0.064
	Canopy size (m^2^)	0.4	4.3	0.002	0.064
	Underbranch height (m)	0.4	4.6	0.004	0.128
Background conditions	Build area (%)	1.3	12.8	0.002	0.064
	Green space area (%)	1.1	12.3	0.002	0.064
	LF forest type	0.8	7.9	0.002	0.064
	Ring road	0.9	9.6	0.002	0.064
	History gradient	0.5	4.9	0.002	0.064
	Mean build distance (m)	0.5	5.6	0.004	0.128
	Build height nearest (m)	0.4	3.8	0.008	0.256
	Water area (%)	0.3	3.6	0.008	0.256
Data statistics	Microclimate conditions	36.3/100			
	Forest characteristics	3.8/10.5			
	Background conditions	5.8/16.0			

To find the differences between the explanatory power of the forest characteristics, weather conditions, and background conditions, we performed summation of the conditional term and simple term effects (Table 2) as well as RDA-related variation partitioning (Fig. 7B). As shown in Table 2, weather condition explained 36.3% (conditional effect) to 58.5% (simple conditional effect) of the variations in microclimate regulation, followed by background conditions (5.8%–15.6%). Forest characteristics had the lowest explanatory power for microclimate regulation (3.8%–9.1%). RDA variation partitioning confirmed these overall findings (Fig. 7B). Unique weather conditions, background conditions, and forest characteristics explained 65.4%, 12.1%, and 7.7% of the microclimate regulation variation observed in this study, respectively. Moreover, interaction among the three groups of parameters showed evident explanatory power for variations in microclimate regulation. For example, the interaction between the background and weather conditions was 11.7%, which was almost the same as the unique background condition contribution (12.1%). Pooled unique and interaction data, together with weather conditions, background conditions, and forest characteristics, explained 78.3%, 25.2% and 10.7% of the variation in microclimate regulation, respectively (Fig. 7B).

## Discussion

### Microclimate regulating functions of urban trees: urban-rural pattern and complexity

In China, the urban green infrastructure is managed according to its location, e.g., downtown or rural regions, and roadside or affiliated forests. The characterization of microclimate regulatory differences at different sites may favor the management of urban forests. Urban-rural patterns of microclimate regulatory functions are not well defined, although urbanization is the largest land use modification that occurs worldwide (Lorenz & Lal, 2015). In this study, through a large field survey of 152 sites in a 500 km^2^ urban region, we found that urban forests provide 77 to 90% sunlight interception, 3 to 4.5 °C of horizontal cooling, 1 to 2 °C of soil cooling, and approximately 1 °C of vertical cooling in the forest canopy. Comparing urban centers to rural regions, we found non-monological changes in five out of the six microclimate regulating parameters that described microclimate regulating functions, which were mainly controlled by weather conditions (*T*_air_, RH, and light intensity) rather than forest characteristics or background conditions of street canyons, land use configuration, and urbanization intensities.

The shading effect of both total intercepted light and relative changes of shading light increased in three urban-rural gradients, indicating a linear decrease in the shading effects from trees during urbanization processes. Some studies reflected the location-dependence of shading effects, for example, streets with a high-percentage of canopy cover had both air temperature, relative humidity, and solar light that were significantly lower than streets with a low percentage of canopy cover (Sanusi et al., 2016). Association analysis of shading effects also indicated that increasing light intensity outside of the urban forests is responsible for the majority of the difference in intercepted light patterns between the urban center and rural regions (Fig. 7A). Therefore, the shading effects were negatively related to street canyon features (distance to buildings) and the tree under-branch height, but positively related to tree density. These strong associations could contribute to the urban-rural shading effect pattern, and this pattern should be fully considered for the maximization of ecological services from urban forests.

Unlike the shading effect, cooling and humidifying effects showed non-monological changes between urban center and rural regions. In semi-arid and semi-moist climate regions such as Changchun, dry air will often cause discomfort in residents. Our results confirmed that the urban forests had clearly observable humidifying effects (<9.4%), and a similar range (3–6%) was reported in Harbin City, which is in the same region as Changchun (Wang et al., 2018b; Zhang et al., 2017a). However, this range was much lower than those reported by Georgi & Zafiriadis (2006) and the 12.4% increase reported by von Von Arx, Dobbertin & Rebetez (2012). Cooling effects from urban trees have been well documented, and our results give a holistic view of these effects in our analysis of horizontal air cooling (3.0–4.5 °C), soil cooling (<1.4 °C) and canopy air cooling (<1.4 °C). Until now, very few studies have focused on vertical cooling from urban trees and forests, although vertical cooling has become more important for urban regions due to building-induced utilization of airspace (Perini et al., 2013; Wang et al., 2018b; Zhang et al., 2017a). The non-monological changes in cooling and humidifying effects from the urban center and rural regions indicate that location-dependent differences in microclimate regulation are not a direct result of urbanization intensity (urban-rural gradient). *T*_air_, RH, and light intensity contributed most of the variation observed in microclimate regulation (>65%). The well-accepted model of the urban heat island generally assumes that *T*_air_ and RH decrease from urban centers to rural regions (Ren et al., 2013). Thus, there are possibly more microclimate regulating functions in urban centers, although no linear changes were observed in cooling and humidifying effects during our study. More exact and simultaneous measurements in different urban regions (e.g., remote sensing; Ren et al., 2013; Zhao et al., 2015) may be needed to understand how these factors contribute to microclimate regulation.

### Reasons for the variations in microclimate regulation

Partitioning the variation in microclimate regulation by urban trees will help define the relative importance of forest characteristics, weather conditions, and background conditions that include buildings, streets, land use configuration, and urbanization intensity. Redundancy analysis (RDA) is a method to define and summarize the relationships between a set of response variables and a set of explanatory variables. More accurately, RDA is a direct gradient analysis technique that summarizes linear functions between components of response variables that are “redundant” or explained by a set of explanatory variables (Legendre & Legendre, 1998; Wang et al., 2017b). Together with our large-scale field survey data, RDA made it possible to decouple the relative explanatory power of microclimate regulatory functions from weather conditions, forest characteristics, and background conditions.

Weather conditions could shape the microclimate regulatory functions of trees (Shashuabar et al., 2010), and this was confirmed by the RDA ordination (simple effects, 58.5%; conditional effects, 36.3%) and variation partitioning (unique contribution, 65.4%; total contribution, 78.3%) reported in this paper. In general, dry, sunny, and hot weather accompanied larger shading, humidifying, and cooling differences. These kinds of findings have been described previously (Jim & Zhang, 2015; Shashuabar et al., 2010; Zhang et al., 2017a), and 2-fold cooling effects were observed on hot, clear days in comparison to cold, cloudy days (Wang et al., 2015).

Our study also found that background conditions made the second largest contribution to variations in microclimate regulation, and the explanatory power of these variables was one-sixth to one-third that of weather conditions (Table 2 and Fig. 7). Street canyon features were highlighted as a contributing factor, indicated by the reduction in *T*_air_ on east–west streets (2.1 °C), which was over 2-fold higher than that measured on north-south streets (0.9 °C) (Sanusi et al., 2016). Moreover, the beneficial cooling of street tree canopies increases as street canyon geometry changes (Coutts et al., 2016).

Forest characteristics had significant explanatory power for variations in microclimate regulation, but displayed the lowest contribution of the variables tested in this study; The contribution of forest characteristics was one-ninth to one-seventh that of weather conditions outside the urban forest. We found tree size-dependent microclimate regulatory functions in this study. Larger tree size was usually accompanied by smaller differences in microclimate regulation between canopy-shaded and open areas, indicating a larger distance of microclimate regulation outside direct tree shade. Similarly, the stronger cooling regulatory functions were also shown by the relatively larger areas experiencing cooling in urban tree communities (Ren et al., 2013; Zhang, Lv & Pan, 2013). Forest parameters previously assessed for their regulatory functions included leaf area index, foliage aggregation, average leaf inclination angle, vertical distribution of foliage (Sampson & Smith, 1993), canopy cover ratio, leaf area index (Yan et al., 2012), leaf angle, leaf size, and canopy architecture or simply canopy density (Sanusi et al., 2017). As a supplement to previous studies, we evaluated the additional parameters of tree height, canopy size, under-branch height, diameter, and tree density, which also played significant roles in microclimate regulation. Furthermore, there was a close association between tree type composition and microclimate regulation, as indicated by the association of stronger cooling and humidifying effects with a higher percentage of Ulmaceae and a lower percentage of Leguminosae, Juglaceae, and Salicaceae. Recent studies have shown similar results, showing that individual trees of *Caesalpinia pluviosa* can reduce air temperature by 12 to 16 °C, which is much higher than other species (Abreu-Harbich, Labaki & Matzarakis, 2015). Denser foliage cover and branching patterns in Calophyllaceae species (*Mesua ferrea*) also functioned as more significant thermal radiation filters than foliage and branching patterns in Euphorbiaceae species (Shahidan et al., 2010). In mahogany (Meliaceae family) plantations, the soil temperature was higher, but the relative humidity was lower than in secondary forests (Lee et al., 2006; Norton et al., 2015). Thus, the RDA ordination results support proper species selection that favors forest microclimate regulation (Abreu-Harbich, Labaki & Matzarakis, 2015; Shahidan et al., 2010).

### Implications: methodology, forest management, and uncertainty

The complex associations between forest characteristics, local weather conditions, background conditions, and multiple kinds of microclimate regulation, should be dissected to improve the efficiency of new forestation practices and management of current forests. Most studies have used specific experimental designs that assume there are no changes across areas or regions. For example, when studying street orientation effects on microclimate regulation, species composition was assumed to be the same on different streets (Sanusi et al., 2016), while species differences in microclimate regulation were compared without accounting for the influence of local weather (Mo et al., 2007; Wang et al., 2015; Zhou et al., 2005). In fact, tree species, weather conditions, and background conditions differed at all surveyed sites, which contradict these generally accepted assumptions. The RDA ordination and variation portioning of large field survey data methodology used in this paper argues for partitioning the relative contribution from different groups of parameters in future studies. This new method can be used in other studies that focus on similar scientific issues both in China and other countries. When used in the study of microclimate regulation, most of the variation could be explained by weather conditions, which were four- to ten-fold times more influential than those from background conditions, including street canyon and land use configuration, and forest characteristics in general (Table 2 and Fig. 7). Thus, the patterns of microclimatic regulation in urban-rural gradients are mainly a result of differences in local weather, rather than urbanization and forest characteristics. Unlike microclimate regulation, changes to biomass and soil carbon sequestration (Lv et al., 2016), and tree species richness and diversity (Xiao et al., 2016b), were a direct result of urbanization intensity itself.

Urbanization could greatly alter resident microclimates, including the urban heat island effect; ameliorating these effects through urban forestry and greening practices is a currently important scientific issue. Owing to the uncontrolled weather conditions present in any city, urban forest management and proper matching of tree types with background conditions are effective ways to improve the urban microclimate. First, a lower percentage of Leguminosae, Juglaceae, and Salicaceae in forests, together with a higher percentage of Ulmaceae, can improve microclimate regulating functions, suggesting that selecting these species in the appropriate proportion can lead to greater microclimate regulating success. Second, the correlation between larger trees and potentially larger cooling and humidifying effect distance indicates that the protection of larger trees could improve the urban microclimate. Changchun city had a low percentage of large trees: only 8% of trees were 15 to 23 m tall, and for the DBH perimeter, only 10% of the trees were large (100–230 cm). Only 9% of trees had a canopy projection area >50 m^2^.

Furthermore, the strong interaction between forest characteristics and background conditions explains variations in microclimate regulation, and suggests the use of different management techniques for urban forests at different urbanization regions in order to maximize the forest ecosystem (Zhang et al., 2017b). Interaction between background conditions and weather conditions explained 11.7% of variations in microclimate regulation and was almost equal to the explanatory power from unique background conditions (12.1%). The interaction between forest characteristics and background conditions also accounted for one-fourth of the explanatory power of forest characteristics (7.7%). Drier and hotter weather usually improve the transpiration rate of tree leaves, which is consistent with the stronger shading and humidifying effects in urban trees (Mo et al., 2007; Yan et al., 2012). Tree size was composition-dependent in Changchun (Table A4). Accordingly, location-dependent forest management practices should be carried out according to the possible interactions between tree size, species composition, and background street and weather conditions.

This research also had limitations. Some causal relationships derived from RDA analysis and variation partitioning possibly represent an over-interpretation of the complex associations (Legendre & Legendre, 1998). Hopefully, previous work has found experimental or modeling methods to assess the importance of background conditions, tree size, and tree configuration (Mo et al., 2007; Shahidan et al., 2010; Shashuabar et al., 2010; Zhao et al., 2015; Zhao, Sailor & Wentz, 2018a). In the future, more detailed work related to tree composition and configuration experiments are needed to test the findings of this paper.

## Conclusions

Multiple microclimate regulatory functions significantly differed at different urban-rural gradients, and only shading effects and total light interception showed a linear increase from the urban center to rural regions. Horizontal cooling was 4.5 °C at most, and the humidifying effect was <9.4%. Vertical cooling effects ranged from 0 to 1.4 °C, while soil cooling was at most 1.4 °C. Redundancy analysis indicates that differences in weather were the major factor responsible for variation in microclimate regulation, followed by background conditions and forest characteristics. Our data provide a basis for location-based urban forest improvement for maximizing the microclimate regulatory functions of these forests.

##  Supplemental Information

10.7717/peerj.5450/supp-1 Data S1 Raw data

10.7717/peerj.5450/supp-2 Supplemental Information 1 Microclimate regulation differences in different ring roads, land uses, and urban history regions

10.7717/peerj.5450/supp-3 Supplemental Information 2 Family-related compositional variations at different ring road regions and land uses

10.7717/peerj.5450/supp-4 Supplemental Information 3 Background differences in RH, *T*_air_, light, and percentage of road, build, green space and water regions (in 200*200 m^2^ squares) at different land uses, urban history regions and ring road regions

10.7717/peerj.5450/supp-5 Supplemental Information 4 Species composition for each family group and their characteristics of tree height, DBH, under branch height and canopy projection size

10.7717/peerj.5450/supp-6 Supplemental Information 5 Example for measuring surrounding background conditions (A) Land use configurations of building, road, green space and water in percentage; (B) Height of nearest building to the measured trees (assumption 2.5m/floor of building).

10.7717/peerj.5450/supp-7 Supplemental Information 6 Urban-rural changes in microclimate regulation at 3 urban-rural gradients of ring road (left), urban history (middle) and forest types (right)

10.7717/peerj.5450/supp-8 Supplemental Information 7 Urban-rural changes in urban tree sizes and density at 3 urban-rural gradients of ring road (left), urban history (middle) and forest types (right)

10.7717/peerj.5450/supp-9 Supplemental Information 8 Urban-rural changes in background conditions at 3 urban-rural gradients of ring road (left), urban history (middle) and forest types (right)

## Additional Information and Declarations

### Data Availability

The following information was supplied regarding data availability: The raw data are provided in a Supplemental File.
