# Supplementary material for: Decoupling forest characteristics and background conditions to explain urban-rural variations of multiple microclimate regulation from urban trees
Source: PeerJ. 2018 Aug 16;6:e5450. doi: 10.7717/peerj.5450 (PMC6098947; doi:10.7717/peerj.5450)
Supplement: Supplemental Information 1 [file peerj-06-5450-s002.docx]

|  | horizontal cooling ΔT_1_℃ | | humidifying effect ΔRH % | | light intercepted ΔE kLux | | vertical cooling ΔT_2_℃ | | soil cooling ΔT_3_℃ | | shading percentage % | |
| --- | --- | --- | --- | --- | --- | --- | --- | --- | --- | --- | --- | --- |
| Ring road urban-rural gradient (ring) | | | | | | | | | | | | |
| 1ringRD | 3.45 | ab | 6.13 | a | 39.2 | a | -0.1 | b | 0.2 | ab | 84.5 | a |
| 2ringRD | 3.06 | a | 5.55 | a | 37.5 | a | -0.7 | ab | 0.5 | b | 82.7 | a |
| 3ringRD | 3.67 | ab | 4.66 | a | 40.1 | a | -1.2 | a | 1.2 | b | 84.2 | a |
| 4ringRD | 4.25 | b | 9.41 | b | 56.5 | ab | -0.6 | ab | 0.5 | b | 85.8 | a |
| Out5ringRD | 3.21 | a | 4.62 | a | 77.1 | b | -1.4 | a | -0.8 | a | 85.5 | a |
| Urban history urban-rural gradient (yr) | | | | | | | |  |  |  |  |  |
| 114-yr | 4.46 | c | 0.46 | a | 31.3 | a | -0.8 | a | -0.5 | a | 83.7 | ab |
| 60-yr | 3.30 | ab | 6.65 | b | 38.6 | a | -0.8 | a | 0.5 | ab | 81.3 | a |
| 24-yr | 2.96 | a | 5.09 | b | 36.7 | a | 0.0 | a | 0.9 | ab | 86.3 | b |
| 10-yr | 4.19 | bc | 4.20 | b | 44.3 | ab | -1.2 | a | 1.4 | b | 84.3 | ab |
| New 0-yr | 3.35 | ab | 4.87 | b | 77.5 | b | -1.3 | a | -0.6 | a | 86.2 | b |
| Land uses-related urban-rural gradient | | | | | | | |  |  |  |  |  |
| AF | 3.85 | a | 6.69 | c | 34.7 | a | -0.7 | a | 1.3 | c | 85.0 | ab |
| RF | 3.51 | a | 3.38 | a | 54.7 | a | -1.4 | a | 0.8 | bc | 86.2 | b |
| LF | 3.16 | a | 5.97 | bc | 34.6 | a | -0.8 | a | -0.2 | a | 82.4 | a |
| EF | 3.43 | a | 4.32 | ab | 95.3 | b | -1.4 | a | 0.0 | ab | 86.5 | b |
