# Supplementary material for: Decoupling forest characteristics and background conditions to explain urban-rural variations of multiple microclimate regulation from urban trees
Source: PeerJ. 2018 Aug 16;6:e5450. doi: 10.7717/peerj.5450 (PMC6098947; doi:10.7717/peerj.5450)
Supplement: Supplemental Information 2 [file peerj-06-5450-s003.docx]

|  | Species relative abundance (%) | | | | | | | | | Tree size and density | | | | | | | | | |
| --- | --- | --- | --- | --- | --- | --- | --- | --- | --- | --- | --- | --- | --- | --- | --- | --- | --- | --- | --- |
|  | Leguminosae % | Betulaceae% | Fagaceae % | Aceraceae % | Rosaceae% | Pinaceae % | Salicaceae% | Ulmaceae % | Otherspp % | Tree Height m | | DBH  cm | | Under branch H m | | Canopy size m^2^ | | Tree density | |
| Ring road (ring) | | | | | |  |  |  |  |  |  |  |  |  |  |  |  |  |  |
| 1ringRD | 4.7 | 10.9 | 3.1 | 3.1 | 6.3 | 46.9 | 15.6 | 3.1 | 6.3 | 10.1 | c | 65.0 | b | 3.6 | c | 29.9 | b | 0.11 | a |
| 2ringRD | 4.4 | 7.3 | 2.2 | 8.0 | 21.2 | 26.3 | 19.7 | 4.4 | 6.6 | 7.9 | b | 52.3 | ab | 2.6 | ab | 26.3 | ab | 0.12 | a |
| 3ringRD | 1.1 | 3.4 | 2.3 | 8.5 | 25.4 | 24.3 | 24.3 | 5.6 | 5.1 | 7.1 | b | 48.0 | a | 2.2 | ab | 20.0 | ab | 0.07 | a |
| 4ringRD | 0.0 | 6.3 | 0.0 | 0.0 | 18.8 | 15.6 | 40.6 | 12.5 | 6.3 | 5.6 | a | 41.1 | a | 2.0 | a | 18.9 | a | 0.08 | a |
| Out4ringRD | 1.0 | 3.1 | 3.1 | 1.5 | 7.7 | 20.0 | 51.3 | 4.6 | 7.7 | 9.7 | c | 64.2 | b | 3.0 | bc | 22.1 | ab | 0.31 | b |
| Urban history (yr) | | | | |  |  |  |  |  |  |  |  |  |  |  |  |  |  |  |
| 114-yr | 0.0 | 20.0 | 10.0 | 10.0 | 0.0 | 25.0 | 25.0 | 10.0 | 0.0 | 9.2 | b | 52.6 | ab | 3.9 | b | 16.2 | a | 0.12 | a |
| 60-yr | 3.6 | 3.6 | 1.6 | 5.7 | 19.8 | 31.3 | 24.0 | 4.7 | 5.7 | 8.3 | ab | 57.8 | ab | 2.6 | a | 26.5 | a | 0.09 | a |
| 24-yr | 2.6 | 5.3 | 2.6 | 10.5 | 29.8 | 22.8 | 15.8 | 5.3 | 5.3 | 7.0 | a | 48.0 | ab | 2.3 | a | 25.2 | a | 0.11 | a |
| 10-yr | 1.5 | 11.8 | 4.4 | 4.4 | 16.2 | 32.4 | 17.6 | 1.5 | 10.3 | 7.3 | a | 44.2 | a | 2.3 | a | 17.0 | a | 0.06 | a |
| New 0-yr | 0.9 | 2.8 | 1.9 | 1.4 | 7.6 | 19.0 | 53.1 | 6.2 | 7.1 | 9.4 | b | 61.9 | b | 3.0 | ab | 21.7 | a | 0.29 | b |
| Forest types | | | |  |  |  |  |  |  |  |  |  |  |  |  |  |  |  |  |
| RF | 0.0 | 1.9 | 3.9 | 10.6 | 17.3 | 20.2 | 40.4 | 1.0 | 4.8 | 7.9 | b | 56.9 | bc | 2.5 | a | 24.2 | a | .08 | ab |
| AF | 2.1 | 6.3 | 2.1 | 2.1 | 35.4 | 18.8 | 13.5 | 12.5 | 7.3 | 6.3 | a | 44.9 | a | 1.9 | a | 22.0 | a | .06 | a |
| LF | 3.4 | 6.8 | 3.4 | 6.0 | 13.6 | 40.0 | 17.0 | 3.8 | 6.0 | 8.8 | bc | 53.9 | ab | 3.3 | b | 23.0 | a | .12 | b |
| EF | 1.4 | 3.6 | 0.0 | 1.4 | 7.9 | 5.7 | 66.4 | 5.7 | 7.9 | 9.4 | c | 65.6 | c | 2.3 | a | 23.4 | a | .37 | c |
