# Supplementary material for: Decoupling forest characteristics and background conditions to explain urban-rural variations of multiple microclimate regulation from urban trees
Source: PeerJ. 2018 Aug 16;6:e5450. doi: 10.7717/peerj.5450 (PMC6098947; doi:10.7717/peerj.5450)
Supplement: Supplemental Information 3 [file peerj-06-5450-s004.docx]

|  | landuse percentage and surrounding differences | | | | | | | | | | | | |  | Weather differences | | | | | |
| --- | --- | --- | --- | --- | --- | --- | --- | --- | --- | --- | --- | --- | --- | --- | --- | --- | --- | --- | --- | --- |
|  | Road-area% |  | Build-area% |  | Greenspace-area% |  | Water-area% |  | Building height nearest to trees m | | Closest distance to trees m | | Mean distance to trees m | | T_air_ ^o^C | | Light kLux | | RH % | |
| Ring road (ring) | |  |  |  |  |  |  |  |  |  |  |  |  |  |  |  |  |  |  |  |
| 1ringRD | 18.1 | b | 11.4 | a | 59.8 | c | 10.8 | b | 22.0 | a | 123.3 | b | 256.9 | b | 31.3 | a | 45.8 | a | 52.5 | a |
| 2ringRD | 19.8 | b | 18.0 | b | 50.5 | b | 11.4 | b | 27.8 | b | 80.6 | ab | 235.9 | b | 31.3 | a | 44.1 | a | 58.5 | bc |
| 3ringRD | 31.1 | c | 22.3 | b | 44.4 | b | 2.3 | a | 35.1 | c | 54.4 | a | 155.7 | a | 30.8 | a | 45.5 | a | 59.7 | c |
| 4ringRD | 46.9 | d | 18.1 | b | 35.8 | a | 1.8 | a | 37.9 | c | 63.3 | ab | 105.6 | a | 31.7 | a | 61.4 | ab | 55.1 | ab |
| Out4ringRD | 11.9 | a | 10.6 | a | 74.7 | d | 2.8 | a | 16.9 | a | 308.6 | c | 657.0 | c | 32.0 | a | 85.2 | b | 55.8 | abc |
| Urban history (yr) | | | | | |  |  |  |  |  |  |  |  |  |  |  |  |  |  |  |
| 114-yr | 26.0 | b | 18.0 | a | 51.0 | a | 5.0 | a | 10.8 | a | 53.6 | a | 133.7 | a | 29.0 | a | 43.0 | a | 60.5 | a |
| 60-yr | 24.2 | b | 17.3 | a | 47.9 | a | 10.4 | b | 29.3 | c | 79.5 | ab | 185.1 | a | 31.5 | b | 45.0 | a | 56.7 | a |
| 24-yr | 25.7 | b | 18.5 | a | 52.8 | a | 3.1 | a | 39.5 | d | 71.3 | ab | 218.9 | a | 30.4 | ab | 41.2 | a | 59.5 | a |
| 10-yr | 29.4 | b | 16.4 | a | 50.2 | a | 4.1 | a | 26.3 | c | 131.9 | ab | 332.7 | b | 32.1 | b | 50.5 | ab | 56.6 | a |
| New 0-yr | 14.7 | a | 13.7 | a | 69.5 | b | 2.5 | a | 18.0 | b | 271.7 | b | 578.4 | c | 31.9 | b | 85.3 | b | 56.4 | a |
| Forest types |  |  |  |  |  |  |  |  |  |  |  |  |  |  |  |  |  |  |  |  |
| RF | 42.0 | c | 25.5 | c | 30.9 | a | 1.5 | a | 38.2 | c | 37.8 | b | 88.9 | a | 31.3 | a | 59.7 | a | 56.7 | a |
| AF | 30.9 | b | 32.5 | d | 36.6 | b | 0.4 | a | 20.4 | a | 17.5 | a | 41.7 | a | 31.6 | a | 39.1 | a | 55.6 | a |
| LF | 15.4 | a | 8.0 | a | 65.9 | c | 10.9 | b | 28.6 | b | 252.2 | d | 587.3 | c | 30.6 | a | 41.2 | a | 58.3 | a |
| EF | 13.0 | a | 14.1 | b | 71.8 | d | 1.0 | a | 17.3 | a | 125.8 | c | 266.3 | b | 33.0 | b | 105.0 | b | 56.7 | a |
