# Supplementary material for: Decoupling forest characteristics and background conditions to explain urban-rural variations of multiple microclimate regulation from urban trees
Source: PeerJ. 2018 Aug 16;6:e5450. doi: 10.7717/peerj.5450 (PMC6098947; doi:10.7717/peerj.5450)
Supplement: Supplemental Information 4 [file peerj-06-5450-s005.docx]

| Family | | Tree height m | DBH perimeter cm | Underbranch height m | Canopy size m^2^ | species name |
| --- | --- | --- | --- | --- | --- | --- |
| Leguminosae | Mean | 8.5 | 61.4 | 3.5 | 27.8 | *Albizia kalkora*; *Gleditsia sinensis* |
|  | SD | 3.5 | 30.6 | 2.3 | 24.4 |  |
| Betulaceae | Mean | 9.5 | 36.7 | 3.2 | 19.2 | *Betula platyphylla* |
|  | SD | 3.1 | 22.0 | 1.3 | 33.0 |  |
| Fagaceae | Mean | 9.5 | 55.9 | 2.3 | 17.0 | *Quercus mongolica*; *Quercus wutaishansea* |
|  | SD | 2.7 | 12.0 | 0.5 | 9.0 |  |
| Aceraceae | Mean | 6.4 | 45.3 | 2.0 | 19.0 | *Acer negundo*; *Acer mono*; *Acer mandshurica*; *Acer ginnala* |
|  | SD | 1.9 | 17.4 | 0.9 | 11.8 |  |
| Rosaceae | Mean | 5.0 | 31.2 | 1.3 | 17.6 | *Amygdalus persica*; *Prunus* spp.; *Armeniaca vulgaris* |
|  | SD | 2.2 | 18.0 | 0.9 | 21.4 |  |
| Pinaceae | Mean | 8.9 | 57.7 | 3.4 | 20.6 | *Pinus* spp.; *Larix* spp. *Picea koraiensis*; *Abies nephrolepis* |
|  | SD | 4.4 | 24.7 | 3.1 | 17.1 |  |
| Salicaceae | Mean | 10.7 | 78.9 | 3.2 | 32.5 | *Populus* spp.; *Salix* spp. |
|  | SD | 4.0 | 43.9 | 2.7 | 29.4 |  |
| Ulmaceae | Mean | 5.1 | 40.2 | 1.9 | 19.1 | *Ulmus pumila* |
|  | SD | 3.2 | 27.9 | 1.2 | 25.2 |  |
| Other spp | Mean | 5.7 | 30.1 | 1.7 | 11.4 | *Tillia* spp., *Franxinus* spp. Juglans spp. Catalpa ovata |
|  | SD | 2.0 | 21.8 | 0.6 | 10.9 |  |
| Total | Mean | 8.4 | 55.7 | 2.7 | 23.1 |  |
|  | SD | 4.2 | 36.0 | 2.4 | 24.2 |  |
