# Supplementary figures and images for: Decoupling forest characteristics and background conditions to explain urban-rural variations of multiple microclimate regulation from urban trees
Source: PeerJ. 2018 Aug 16;6:e5450. doi: 10.7717/peerj.5450 (PMC6098947; doi:10.7717/peerj.5450)

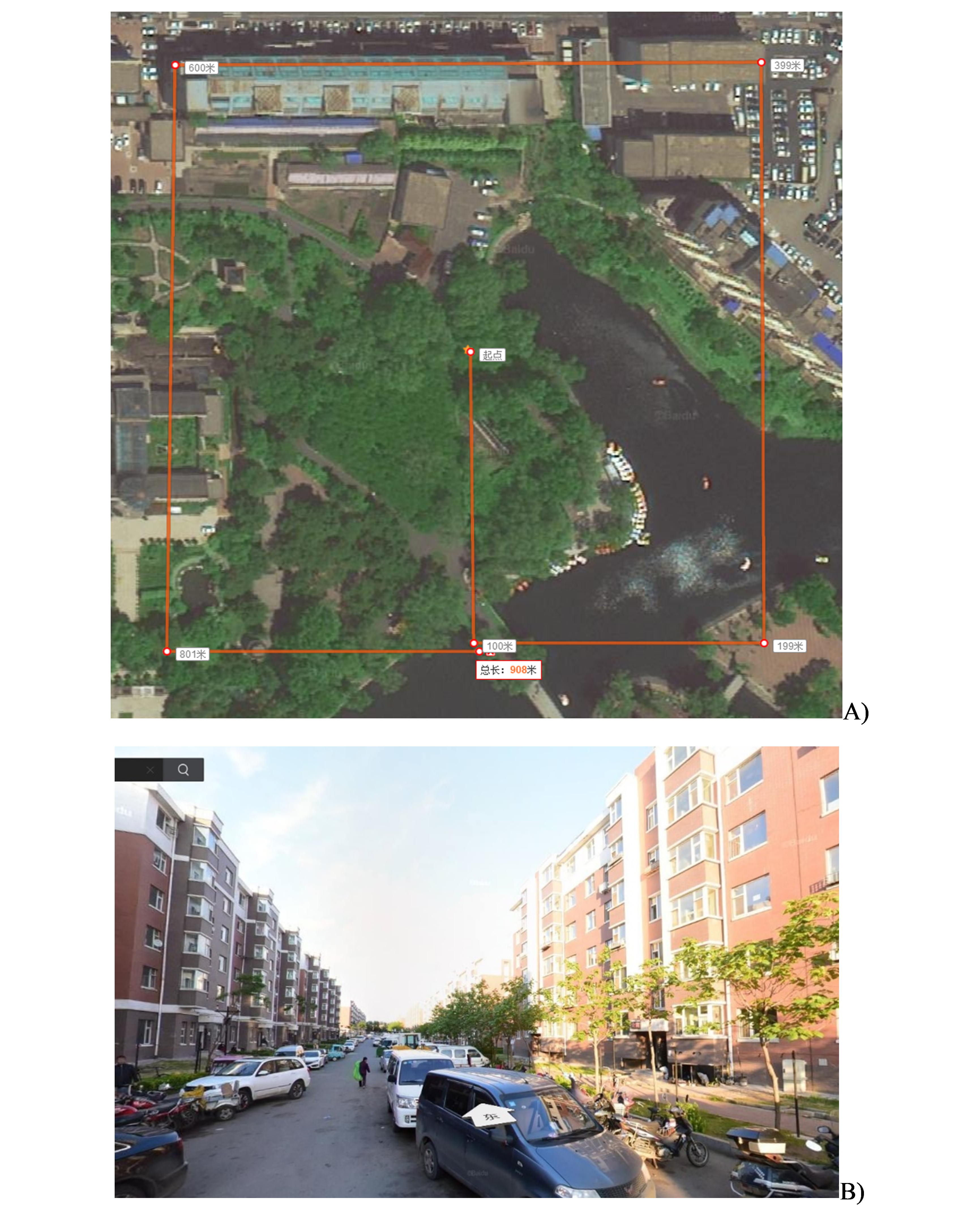

Supplement: Supplemental Information 5 — (A) Land use configurations of building, road, green space and water in percentage; (B) Height of nearest building to the measured trees (assumption 2.5m/floor of building). [file peerj-06-5450-s006.png]

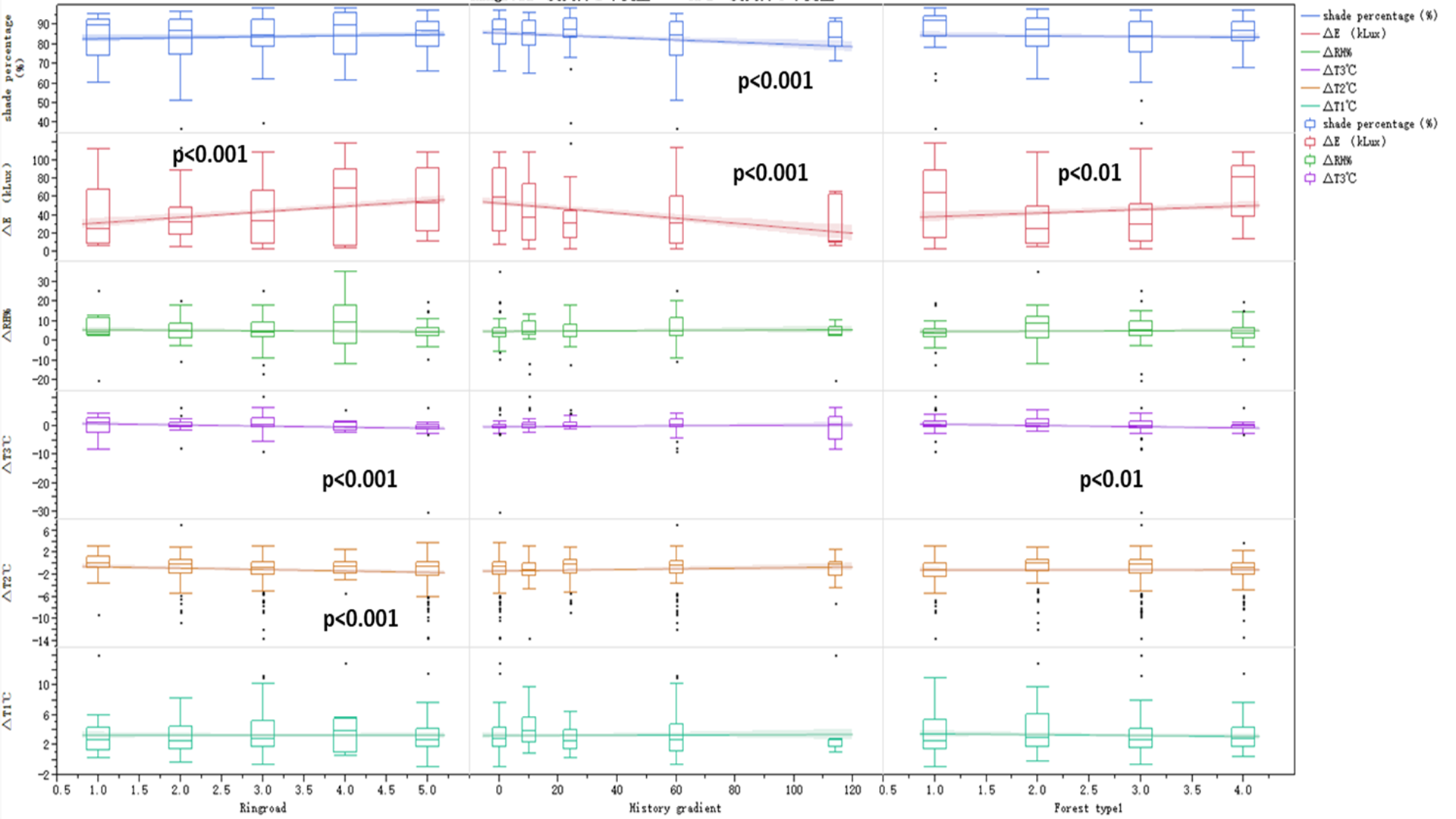

Supplement: Supplemental Information 6 [file peerj-06-5450-s007.png]

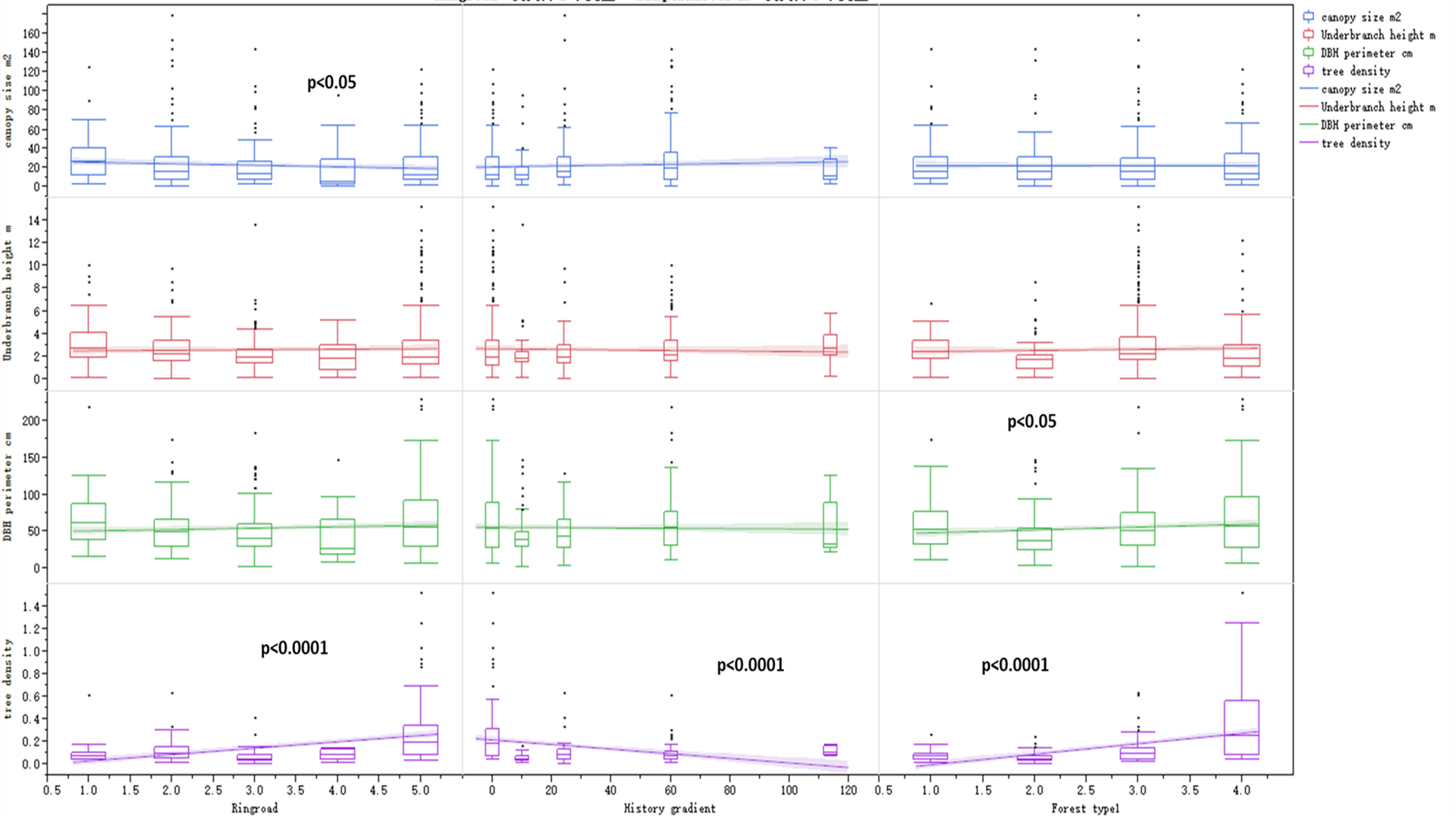

Supplement: Supplemental Information 7 [file peerj-06-5450-s008.png]

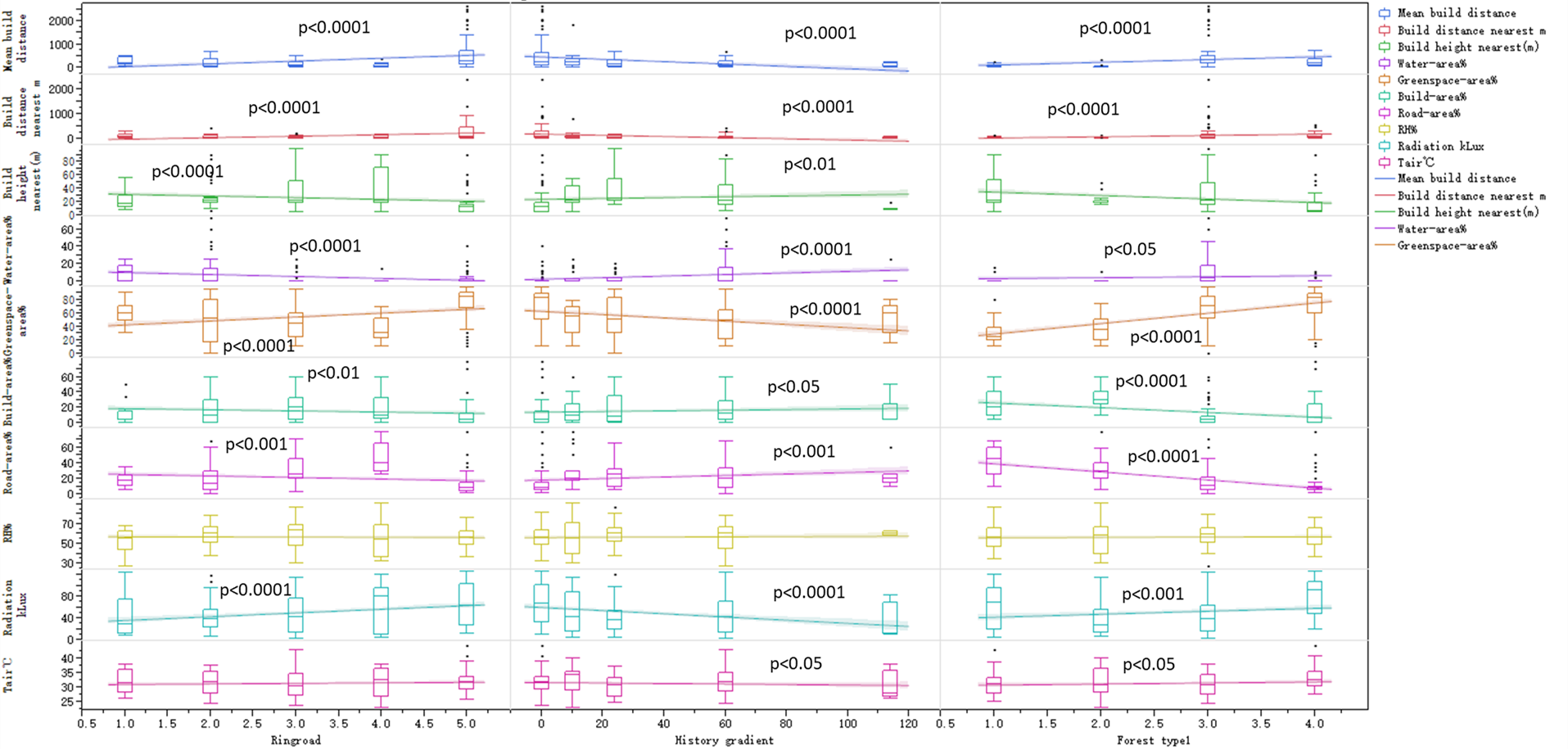

Supplement: Supplemental Information 8 [file peerj-06-5450-s009.png]
